# Supplementary material for: Systematic Review and Meta-Analysis of Integrated Studies on Salmonella and Campylobacter Prevalence, Serovar, and Phenotyping and Genetic of Antimicrobial Resistance in the Middle East—A One Health Perspective
Source: Antibiotics (Basel). 2022 Apr 19;11(5):536. doi: 10.3390/antibiotics11050536 (PMC9137557; doi:10.3390/antibiotics11050536)
Supplement: Supplementary file 1 [file antibiotics-11-00536-s001.zip › Supplementary Table S1. The search strategy used for each database.pdf]

Supplementary Table S1: The search strategy used for each database.

Database	Full search strategy
**PubMed**	Poultry OR Chicken OR “Broiler chicken” OR meat OR Animal OR Livestock OR Human AND Campylobacter OR Salmonella OR “ non-typhoid Salmonella Enterica” AND antimicrobial resistance OR"antimicrobial susceptibility" OR "antibiotic resistance" OR resistome OR AMR OR "Multidrug Resistance" OR Serotype OR prevalence OR "prevalence rate" AND Middle East OR Eastern Mediterranean OR Near East OR EMRO OR Mediterranean Countries OR "Asia" OR Qatar OR United Arab Emirates OR Bahrain OR Saudi Arabia OR Kuwait OR Israel OR Oman OR Iran OR Jordan OR Lebanon OR Palestine OR "West Bank" OR "Gaza Strip" OR Syria OR Yemen OR Turkey OR Iraq OR Egypt OR “North Africa” OR “Africa”. AND (("2010"[Date - Publication]<br>**Timespan:** January 1st, 2010, until September 30th, 2021
**Web of Science**	- **TITLE:** (- ("antimicrobial resistance" OR "antibiotic resistance" OR "antimicrobial susceptibility" OR "Resistome")) AND Salmonella Enterica OR “ non-typhoidal Salmonella Enterica” OR Campylobacter *AND* Poultry OR “Broiler chicken” *OR* Meat *OR* Animals OR Human *OR* **TOPIC:** ((Middle East OR“ East Mediterranean” OR “ Near East” OR EMRO OR Middetrenian Countries OR Asia OR Qatar OR United Arab Emirates OR Bahrain OR Saudi Arabia OR Kuwait OR Israel OR Oman OR Iran OR Jordan OR Lebanon OR Palestine OR "West Bank" OR "Gaza Strip" OR "Occupied Palestinian Territory" OR Syria OR Yemen OR Turkey OR Iraq OR Egypt OR “North Africa” OR “Africa.”<br>- **Timespan:** January 1st, 2010, until September 30th, 2021.
**Scopus**	- (TITLE-ABS-KEY (*"antimicrobial resistance"* OR *"antibiotic resistance"* OR *"antimicrobial susceptibility"* OR *"Resistome"*) AND Salmonella Enterica OR “ non-typhoidal Salmonella Enterica” OR Campylobacter AND Poultry OR “Broiler chicken” AND Meat AND Animals AND Human *AND* TITLE-ABS-KEY (*Middle East OR“ East Mediterranean” OR “ Near East” OR EMRO OR Middetrenian Countries OR Asia OR Qatar OR United Arab Emirates OR Bahrain OR Saudi Arabia OR Kuwait OR Israel OR Oman OR Iran OR Jordan OR Lebanon OR Palestine OR "West Bank" OR "Gaza Strip" OR "Occupied Palestinian Territory" OR Syria OR Yemen OR Turkey OR Iraq OR Egypt OR “North Africa” OR “Africa”*) And<br>AND DOCTYPE (*ar* OR *re*) AND PUBYEAR > *2010*
