# Supplementary material for: Systematic Review and Meta-Analysis of Integrated Studies on Salmonella and Campylobacter Prevalence, Serovar, and Phenotyping and Genetic of Antimicrobial Resistance in the Middle East—A One Health Perspective
Source: Antibiotics (Basel). 2022 Apr 19;11(5):536. doi: 10.3390/antibiotics11050536 (PMC9137557; doi:10.3390/antibiotics11050536)
Supplement: Supplementary file 1 [file antibiotics-11-00536-s001.zip › Supplementary Table S2. Overview of the selected studies.pdf]

Supplementary Table S2. Overview of the selected studies.

ID	Refrence	Country	Pathogen	study design	Source	outcomes of interest	Human Sample size	Animal Sample size
**125**	Aouf 2011; Resistance to -lactams of human and veterinary Salmonella isolates in Egypt and Algeria	Egypt	Salmonella spp.	Routine data-	Human with diarrhea, poultry	1-Nontyphoidal salmonellae_ AMR phenotypes<br>2-Nontyphoidal salmonellae-AMR genotypes	20 isolates	**20 isolates**
**1056**	Youssef 2021; Serotyping and Antimicrobial Resistance Profile of EntericNontyphoidalSalmonellaRecovered from Febrile NeutropenicPatients and poultry in Egypt	Egypt	Salmonella spp.	Cross Sectional	Human with diarrhea, poultry	1- Nontyphoidal salmonellae. Serotypes with prevelance rate.<br>2-Nontyphoidal salmonellae. AMR phenotypes<br>4- AMR genotypes	300	**50 isolates**
**109**	Ammar 2021; Prevalence, Antimicrobial Susceptibility, Virulence and Genotyping of Campylobacter jejuni with a Special Reference to the Anti-Virulence Potential of Eugenol and Beta-Resorcylic Acid on Some Multi-Drug Resistant Isolates in Egypt	Egypt	Campylobacter jejuni	Cross Sectional	Human with diarrhea (meat workers), poultry	1- C Jejuni. prevelance rate<br>2- C Jejuni. AMR phenotypes	100	**245**
**634**	Maysa 2015; Diversity and virulence associated genes of Salmonella enterica serovars isolated from wastewater agricultural drains, leafy green producing farms, cattle and human along their courses	Egypt.	Salmonella spp.	Cross Sectional	Human (Cattle Farm workers), Cattle	1-Salmonella prevelance rate<br>2-Salmonella serotypes	45	**52**

**376**	Gharieb 2015; Non-Typhoidal Salmonella in poultry meat and diarrhoeic patients: prevalence, antibiogram, virulotyping, molecular detection and sequencing of class I integrons in multidrug resistant strains	Egypt	Salmonella spp.	Cross Sectional	Human with diarrhea, poultry meat	1- Nontyphoidal salmonellae. prevelance rate 2- Nontyphoidal salmonellae serotypes 3-Nontyphoidal salmonella AMR phenotype 4- Nontyphoidal salmonellae AMR genotype	50	**50**
**381**	Ghoneim 2021; Isolation and molecular characterization of Campylobacter jejunifrom poultry and human stool samples in Egypt	Egypt	Campylobacter jejuni	Cross Sectional	Human with dirrhea and with contact with animal), poultry	Campylobacter jejuni prevelance rate	75	**360**
**683**	Morshed 2010; Drug resistance, plasmid profile and random amplified polymorphic DNA analysis of Iranian isolates of Salmonella Enteritidis	Iran	Salmonella Enteritidis	Routine Data	Human with diarrhea, poultry and Cattle.	Salmonella Enteritidis_ AMR phenotype	9 isolates	**40 isolates**
**1361**	El-Tras 2015; Campylobacter infections in children exposed to infected backyard poultry in Egypt	Egypt	Campylobacter spp.	Cross Sectional	Children (Healthy, own poultry), poultry	Campylobacter prevelance rate Campylobacter serotypes	106	**379**
**1390**	Firoozeh 2012; Characterization of class I integrons among Salmonella enterica serovar Enteritidis isolated from humans and poultry	Iran	Salmonella Enteritidis	Routine data	Human with diarrhea, poultry	1- Salmonella Enteritidis AMR phenotype 2- AMR genotype	58 isolates	**50 isolates**

**1419**	Ghoddusi 2019; Serotype Distribution and Antimicrobial Resistance of Salmonella Isolates in Human, poultry, and Cattle in Iran	Iran	Salmonella spp.	Routine data	Human with diarrhea, poultry and Cattle	1- Salmonella serotypes and prevelance rates. 2-Salmonella AMR phenotype	109 isolates	**133 isoaltes**
**2353**	Mohamed 2011; Fecal Shedding of Non-typhoidal Salmonella Species in Dairy Cattle and their Attendants in Alexandria Suburbs	Egypt	Salmonella spp.	Cross Sectional	Human (Dairy Farms attendants), Cattle	1- Salmonella Nontyphoidal salmonellae prevelance rate 2- Salmonella Nontyphoidal salmonellae Serotypes 3-Salmonella phenotype	12	**450**
**1678**	Mohammed 2019; The prevalence of Campylobacter species in broiler flocks and their environment: assessing the efficiency of chitosan/zinc oxide nanocomposite for adopting control strategy	Egypt	Campylobacter coli	Cross Sectional	Human (poultry frams workers), poultry farms	Campylobacter. coli prevelance rate	30	**160**
**1090**	AbdEl-Aziz 2020; First Report of aacC5-aadA7Δ4 Gene Cassette Array and Phage Tail Tape Measure Protein on Class 1 Integrons of Campylobacter Species Isolated from Animal and Human Sources in Egypt	Egypt	Campylobacter spp.	Cross Sectional	Human with diarrhea, poultry, meat, milk, and milk products	1- Campylobacter prevelance rate 2- Campylobacter serotypes 3-Campylobacter AMR phenotype 4- Campylobacter AMR genotype	30	**520**
**55**	Ahmed 2020; Phylogenetic analysis of Salmonella species isolated from cows, buffaloes, and humans based on gyrB gene sequences	Egypt	Salmonella spp.	Cross Sectional	Human (healthy workers, and children with diarrhea), buffaloes and cows (milk and feces).	Salmonella prevelance rate and serotypes.	160	**800**

**17**	Abdelmalek 2019; Occurrence of Salmonella infection and antimicrobial susceptibility for local Salmonella isolates from different sources in a cross-sectional study	Egypt	Salmonella spp.	Cross Sectional	Human with diarrhea, grilled poultrys	1- Salmonella Nontyphoidal salmonellae prevelance rate 2- Salmonella Nontyphoidal salmonellae serotypes 3- Salmonella Nontyphoidal salmonellae AMR phenotype	75	**50**
**301**	El-Naenaeey 2021; Prevalence and antibiotic resistance patterns of Campylobacter species isolated from different sources in Egypt	Egypt.	Campylobacter spp.	Cross Sectional	Human (gastroenteritis patients), Polutry and cattle	1- Campylobacter prevelance rate and serotypes 2- Campylobacter AMR phenotype	44	**242**
**497**	Jaradat 2014; Comparative analysis of virulence and resistance profiles of Salmonella Enteritidis isolates from poultry meat and foodborne outbreaks in northern Jordan	Jordan	Salmonella Enteritidis	Cross Sectional	Human, poultry meat	1-Salmonella Enteritidi prevelance rate 2- Salmonella Enteritidi _ AMR phenotype	7 isolates	**302**
**467**	Ibrahim 2019; Prevalence, antimicrobial resistance and risk factors for campylobacteriosis in Lebanon	Lebanon	Campylobacter spp.	Cross Sectional	Human with diarrhea, meat (poultry, beef, lamb, and goat)	1- Campylobacter spp prevelance rate and serotypes 2- Campylobacter AMR phenotype	1000	**150**
**3**	Abay 2014; Genetic diversity and antibiotic resistance profiles of Campylobacter jejuniisolates from poultry and humans in Turkey	Turkey	Campylobacter jejuni	Cross Sectional-used Routine data	Human with diarrhea, poultry carcasses	Campylobacter jejuni AMR phenotype	100 isolates	**100 isolates**

**263**	Divsalar 2019; Antimicrobial resistances, and molecular typing of Campylobacter jejuniisolates, separated from food-producing animals and diarrhea patients in Iran	Iran	Campylobacter jejuni	Cross sectional	Human with diarrhea, poultry and cattle (stool sample and meat)	1 Campylobacter jejuni prevelance rate<br>2- Campylobacter jejuni AMR phenotype<br>3- Campylobacter jejuni AMR genotype	74	**233**
**54**	Ahmed 2016; Characterization of Virulence-Associated Genes, Antimicrobial Resistance Genes, and Class 1 Integrons in Salmonella enterica serovar Typhimurium Isolates from poultry meat and Humans in Egypt	Egypt	Salmonella typhimurium	Cross sectional	pluck-shop markets workers, poultry meat	1 Salmonella Typhimurium prevelance rate<br>2-Salmonella typhimurium AMR phenotype<br>3- Salmonella typhimurium AMR genotype	100	**500**
**1839**	Rokney 2020; WGS-Based Prediction and Analysis of Antimicrobial Resistance in Campylobacter jejuniIsolates From Israel	Israel	Campylobacter jejuni	Routine data	Human clinical specimens, poultry and cattle	1- Campylobacter.jejuni AMR phenotype<br>2- Campylobacter jejuni AMR genotype	239 isolates	**24 isolates**
**401**	Greige 2019; Prevalence and genetic diversity of Campylobacter spp. in the production chain of broiler poultrys in Lebanon and its association with the intestinal protozoan Blastocystis sp	Lebanon	Campylobacter spp.	Cohort and Cross sectional	Human (farm workers, vet, and diarrhea patient), poultry meat (Farms and slaughterhouses)	1- Campylobacter prevelance rate<br>2-Campylobacter serotypes	100	**454**

**421**	Hamidian 2011; Prevalence of putative virulence markers in Campylobacter jejuniand Campylobacter coli isolated from hospitalized children, raw poultry, and raw beef in Tehran, Iran	Iran	Campylobacter spp.	Cross sectional	Human (hospitalized children), meat (poultry and beef).	Campylobacter prevelance rate	420	**378**
**383**	Ghoneim 2017; Zoonotic Importance of Campylobacter jejuniIsolated From poultry Farms in Egypt	Egypt	Campylobacter jejuni	Cross sectional	Human (workers at poultry farms and shops), poultry	Campylobacter jejuni prevelance rate	20	**200**
**382**	Ghoneim 2020; Campylobacter Species Isolated from poultrys in Egypt: Molecular Epidemiology and Antimicrobial Resistance	Egypt	Campylobacter jejuni	Cross sectional	Human (workers at poultry farms and shops), poultry	1-Campylobacter prevelance rate<br>2- Campylobacter serotypes<br>3-Campylobacter AMR phenotype<br>4- Campylobacter AMR genotype	50	**360**
**375**	Gharieb 2019; Antibiogram, virulotyping and genetic diversity of Escherichia coli and Salmonella serovars isolated from diarrheic calves and calf handlers	Egypt	Salmonella spp.	Cross Sectional	human (hand swab from calf handlers), Calves with diarrhea	1-Salmonella Nontyphoidal AMR phenotype<br>2- Salmonella Nontyphoidal serotype	35	**80**
**1066**	Zeinhom 2021; Prevalence, Characterization, and Control of Campylobacter jejuniIsolated from Raw milk, Cheese, and Human Stool Samples in Beni-Suef Governorate, Egypt	Egypt	Salmonella spp.	Cross sectional	Human with diarrhea, raw milk and cheese	1- Campylobacter jejuni prevelance rate<br>2- Campylobacter jejuni AMR phenotype	53	**200**

**147**	Awad 2020; Phenotypes, antibacterial-resistant profile, and virulence-associated genes of Salmonella serovars isolated from retail poultry meat in Egypt	Egypt	Salmonella spp.	Cross sectional	Human (retail workers), poultry carcasses	1-Salmonella serotypes and prevelance rate 2-Salmonella AMR phenotype	25	**200**
**10**	AbdEl-Hamid 2019; Genetic Diversity of Campylobacter jejuniIsolated From Avian and Human Sources in Egypt	Egypt.	Campylobacter jejuni	Cross Sectional	Human with diarrhea, poultry (meat, and Cloacal Swab) and pigeon dropletSalmonella	Campylobacter jejuni prevelance rate	270	**270**
**155**	Barakat 2020; Prevalence, molecular detection, and virulence gene profiles of Campylobacter species in humans and foods of animal origin	Egypt	Campylobacter spp.	Cross sectional	Human with diarrhea Human incontact with poultry, poultry, milk and milk products	1- Campylobacter spp prevelance rate 2- Campylobacter serotypes	105	**1194**
**53**	Ahmed 2015; ERIC-PCR Genotyping of Some Campylobacter jejuniIsolates of poultry and Human Origin in Egypt	Egypt	Campylobacter jejuni	Cross sectional	Human with diarrhea, poultry	Campylobacter jejuni prevelance rate	246	**287**
**1234**	Besharati 2020; Serogroups, and drug resistance of nontyphoidal Salmonella in symptomatic patients with community-acquired diarrhea and poultry meat samples in Tehran	Iran	Salmonella spp.	Cross sectional	Human with diarrhea, meat (poultry)	1- Salmonella Nontyphoidal prevelance rate 3- Salmonella AMR phenotype 4- Salmonella AMR genotype	400	**100**
**337**	Farahani 2018; Molecular Detection, Virulence Genes, Biofilm Formation, and Antibiotic	Iran	Salmonella Enteritidis	Routine data	Human, meat (poultry)	Salmonella enteritidis AMR phenotype	36 isolates	**195 isolates**

	Resistance of Salmonella enterica Serotype enteritidis Isolated from poultry and diarrhea Samples							
**76**	Al-Dawodi 2012; Antimicrobial resistance in non-typhi Salmonella enterica isolated from humans and poultry in Palestine	Palestine	Salmonella spp.	Routine data	Children, poultry	Nontyphoidal salmonellae AMR phenotype	71 isolates	**80 isolates**
**1310**	Derakhshandeh 2013; Association of three plasmid-Encoded spv Genes Among Different Salmonella Serotypes Isolated from Different Origins	Iran	Salmonella spp.	Routine data	Human with diarrhea, poultry	Salmonella Nontyphoidal serotypes	5 isolates _	**30 isolates _**
**296**	Elhariri 2020; Virulence and Antibiotic Resistance Patterns of Extended-Spectrum Beta-Lactamase-Producing Salmonella enterica serovar Heidelberg Isolated from Broiler poultrys and poultry Workers: A Potential Hazard	Egypt	Salmonella Heidelberg	Cross Sectional	poultry workers, poultry with diarrhea	1- Salmonella Heidelberg prevelance rate 2-Salmonella Heidelberg.AMR pühenotype 3- Salmonella Heidelberg AMR genoptype	60	**300**
**2175**	Elsayed 2020; Tracing of salmonella contaminations throughout an integrated broiler production chain in Dakahlia Governorate, Egypt	Egypt	Salmonella spp.	Cross sectional	Human (poultry Farms workers), poultry farms	Salmonella prevelance rate and serotypes	15	**395**

**685**	Mouftah 2021; High-throughput sequencing reveals genetic determinants associated with antibiotic resistance in Campylobacter spp. from farm-to-fork	Egypt	Campylobacter spp.	Routine data	Human with diarrhea, poultry carcasses, milk and dairy product	1-Campylobacter AMR phenotype 2- Campylobacter AMR genotype	57 isolates	**54 isolates**
**25**	Acar 2017; Phenotyping and genetic characterization of Salmonella enterica isolates from Turkey revealing arise of different features specific to geography	Turkey	Salmonella spp.	Cross sectional	Human with diarrhea, bovine, ovine, goat, and poultry	1- Salmonella Nontyphoidal salmonellae prevelance rate 2- S serotypes 3-Salmonella AMR phenotype 4- Salmonella AMR genotype	50	**335**
**380**	Ghoneim 2017; Camel as a transboundary vector for emerging exotic Salmonella serovars	Egypt	Salmonella spp.	Cross sectional	Human (slaughterhouse workers), Camel	Nontyphoidal salmonellae prevelance rate	50	**206**
