# Supplementary material for: Systematic Review and Meta-Analysis of Integrated Studies on Salmonella and Campylobacter Prevalence, Serovar, and Phenotyping and Genetic of Antimicrobial Resistance in the Middle East—A One Health Perspective
Source: Antibiotics (Basel). 2022 Apr 19;11(5):536. doi: 10.3390/antibiotics11050536 (PMC9137557; doi:10.3390/antibiotics11050536)
Supplement: Supplementary file 1 [file antibiotics-11-00536-s001.zip › Supplementary Table S3. Summary of the selected studies showing the country and pathogens together.pdf]

Supplementary Table S3. Summary of the selected studies showing the country and pathogens together.

	Country							
**Pathogens**	**Egypt**	**Iran**	**Turkey**	**Lebanon**	**Palestine**	**Jordan**	**Israel**	**Total**
***Salmonella* spp**	**12**	**3**	**1**	**0**	**1**	**0**	**0**	**17**
***S.* Heidelberg**	**1**	**0**	**0**	**0**	**0**	**0**	**0**	**1**
***S.* Typhimurium**	**1**	**0**	**0**	**0**	**0**	**0**	**0**	**1**
S. Enteritidis	**0**	**3**	**0**	**0**	**0**	**1**	**0**	**4**
***Campylobacter* spp**	**5**	**1**	**0**	**2**	**0**	**0**	**0**	**8**
C. jejuni	**6**	**1**	**1**	**0**	**0**	**0**	**1**	**9**
C. coli	**1**	**0**	**0**	**0**	**0**	**0**	**0**	**1**
**Total**	**26**	**8**	**2**	**2**	**1**	**1**	**1**	**41**
